# Naphthalen-1-aminium chloride

**DOI:** 10.1107/S1600536811032569

**Published:** 2011-08-17

**Authors:** Mohammad T. M. Al-Dajani, Hassan H. Adballah, Nornisah Mohamed, Madhukar Hemamalini, Hoong-Kun Fun

**Affiliations:** a School of Pharmaceutical Sciences, Universiti Sains Malaysia, 11800 USM, Penang, Malaysia; b School of Chemical Sciences, Universiti Sains Malaysia, 11800 USM, Penang, Malaysia; c X-ray Crystallography Unit, School of Physics, Universiti Sains Malaysia, 11800 USM, Penang, Malaysia

## Abstract

In the crystal structure of the title compound, C_10_H_10_N^+^·Cl^−^, the two components are connected *via* N—H⋯Cl hydrogen bonds, forming a layer parallel to the *bc* plane.

## Related literature

For applications of naphthalene, see: Griego *et al.* (2008 ▶). For a related structure, see: Pitchumony & Stoeckli-Evans (2005 ▶).
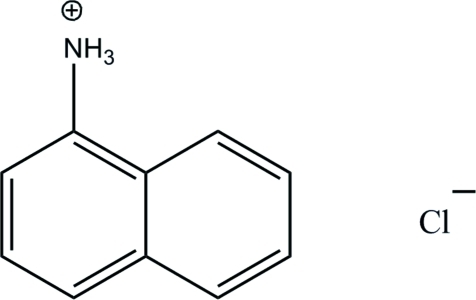


## Experimental

### 

#### Crystal data

C_10_H_10_N^+^·Cl^−^
*M* *_r_* = 179.64
Monoclinic, 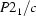

*a* = 13.9691 (11) Å
*b* = 5.2811 (4) Å
*c* = 12.164 (1) Å
β = 93.791 (2)°
*V* = 895.40 (12) Å^3^
*Z* = 4
Mo *K*α radiation
μ = 0.37 mm^−1^
*T* = 296 K
0.50 × 0.11 × 0.06 mm

#### Data collection

Bruker APEXII DUO CCD area-detector diffractometer
Absorption correction: multi-scan (*SADABS*; Bruker, 2009 ▶) *T*_min_ = 0.838, *T*_max_ = 0.978
7193 measured reflections
2612 independent reflections
2013 reflections with *I* > 2σ(*I*)
*R*_int_ = 0.024

#### Refinement

*R*[*F* ^2^ > 2σ(*F* ^2^)] = 0.036
*wR*(*F* ^2^) = 0.100
*S* = 1.05
2612 reflections
110 parameters
H-atom parameters constrained
Δρ_max_ = 0.27 e Å^−3^
Δρ_min_ = −0.17 e Å^−3^

### 

Data collection: *APEX2* (Bruker, 2009 ▶); cell refinement: *SAINT* (Bruker, 2009 ▶); data reduction: *SAINT*; program(s) used to solve structure: *SHELXTL* (Sheldrick, 2008 ▶); program(s) used to refine structure: *SHELXTL*; molecular graphics: *SHELXTL*; software used to prepare material for publication: *SHELXTL* and *PLATON* (Spek, 2009 ▶).

## Supplementary Material

Crystal structure: contains datablock(s) global, I. DOI: 10.1107/S1600536811032569/is2764sup1.cif

Structure factors: contains datablock(s) I. DOI: 10.1107/S1600536811032569/is2764Isup2.hkl

Supplementary material file. DOI: 10.1107/S1600536811032569/is2764Isup3.cml

Additional supplementary materials:  crystallographic information; 3D view; checkCIF report

## Figures and Tables

**Table 1:** Hydrogen-bond geometry (Å, °)

*D*—H⋯*A*	*D*—H	H⋯*A*	*D*⋯*A*	*D*—H⋯*A*
N1—H1*A*⋯Cl1^i^	0.89	2.41	3.1824 (11)	145
N1—H1*B*⋯Cl1^ii^	0.89	2.27	3.1355 (11)	164
N1—H1*C*⋯Cl1	0.89	2.24	3.1225 (11)	170

Symmetry codes: (i) 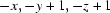; (ii) 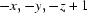.

**Fig. 1.**
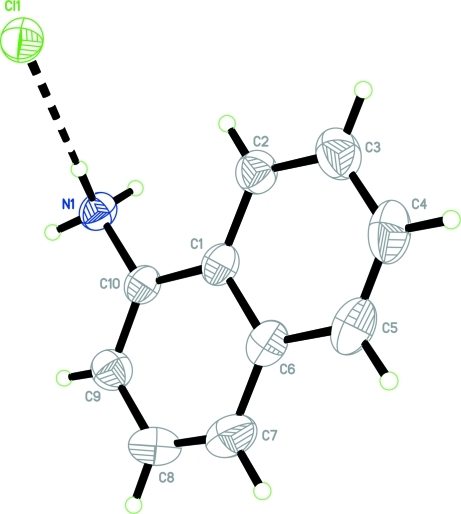
The asymmetric unit of the title compound, showing 50% probability displacement ellipsoids and the atom-numbering scheme. The intermolecular N—H···Cl hydrogen bond shown by a dashed line.

**Fig. 2.**
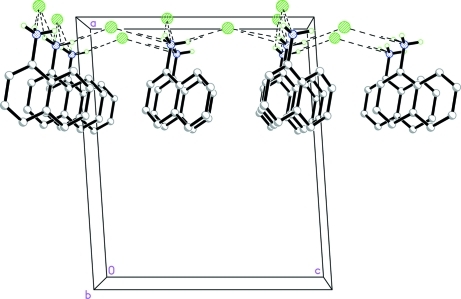
The crystal packing of title compound (I), dashed lines represents hydrogen bonds. H atoms not involved in the hydrogen bond interactions are omitted for clarity.

## supplementary crystallographic information

### Comment

Naphthalene, a bicyclic aromatic compound, can be found environmentally as a constituent of coal tar, crude oil, and cigarette smoke. It is also used in chemical manufacturing as a chemical intermediate for many commercial products ranging from pesticides to plastics. Because of its widespread human exposure (Griego *et al.*, 2008), its toxicological properties have been the subject of numerous assessments. Of particular interest was an evaluation for the potential to induce tumors.  Herein, we have present the crystal structure of napthalen-1-ammonium chloride (I).

The asymmetric unit  of title compound (I), consists of a protonated napthalen-1-ammonium cation and a chloride anion as shown in Fig. 1. In the cation, the C1–C6 bonds are long [1.4260 (17) Å], while the C7–C8, C9–C10, C2—C3 and C4–C5 bonds are short, ranging from 1.357 (3) to 1.370 (2) Å . The remainder of the bonds, C8–C9, C1–C2, C1–C10, C3–C4, C5–C6, C6–C7 and C8–C9 have an intermediate length, ranging from 1.407 (2) to 1.420 (2) Å. This variation indicates that the π electrons are not fully delocalized over the whole nucleus of the naphthalene ring. This situation is similar to that observed in the crystal structure of napththalene 2,3-dicarbonitrile (Pitchumony & Stoeckli-Evans, 2005). The naphthalene ring is essentially planar, with a maximum deviation of 0.007 (2) Å for atom C2. In the crystal structure, (Fig. 2), the ion pairs are connected *via* N—H···Cl hydrogen bonds (Table 1) forming layers parallel to the *bc*-plane.

### Experimental

In a round bottom flask, 25ml of toluene was mixed with 1-nitronapthalene (0.01 mol, 1.5 g) with stirring. Iron powder (0.2 g) dissolved with 5 ml of hydrochloric acid was then added. The mixture was neutralized with sodium hydroxide solution. The blue precipitate formed was washed with alkaline water and then was dissolved in methanol at room temperature. After few days, blue needle-shaped crystals was formed by slow evaporation.

### Refinement

All hydrogen atoms were positioned geometrically (N—H = 0.89 Å and C—H = 0.93 Å) and were refined using a riding model, with *U*_iso_(H) = 1.2*U*_eq_(C).

### Crystal data

**Table:**

C_10_H_10_N^+^·Cl^−^	*F*(000) = 376
*M**_r_* = 179.64	*D*_x_ = 1.333 Mg m^−^^3^
Monoclinic, *P*2_1_/*c*	Mo *K*α radiation, λ = 0.71073 Å
Hall symbol: -P 2ybc	Cell parameters from 2184 reflections
*a* = 13.9691 (11) Å	θ = 2.4–29.5°
*b* = 5.2811 (4) Å	µ = 0.37 mm^−^^1^
*c* = 12.164 (1) Å	*T* = 296 K
β = 93.791 (2)°	Needle, blue
*V* = 895.40 (12) Å^3^	0.50 × 0.11 × 0.06 mm
*Z* = 4	

### Data collection

**Table:**

Bruker APEXII DUO CCD area-detector diffractometer	2612 independent reflections
Radiation source: fine-focus sealed tube	2013 reflections with *I* > 2σ(*I*)
graphite	*R*_int_ = 0.024
φ and ω scans	θ_max_ = 30.0°, θ_min_ = 2.9°
Absorption correction: multi-scan (*SADABS*; Bruker, 2009)	*h* = −19→19
*T*_min_ = 0.838, *T*_max_ = 0.978	*k* = −7→7
7193 measured reflections	*l* = −17→17

### Refinement

**Table:**

Refinement on *F*^2^	Primary atom site location: structure-invariant direct methods
Least-squares matrix: full	Secondary atom site location: difference Fourier map
*R*[*F*^2^ > 2σ(*F*^2^)] = 0.036	Hydrogen site location: inferred from neighbouring sites
*wR*(*F*^2^) = 0.100	H-atom parameters constrained
*S* = 1.05	*w* = 1/[σ^2^(*F*_o_^2^) + (0.0446*P*)^2^ + 0.1614*P*] where *P* = (*F*_o_^2^ + 2*F*_c_^2^)/3
2612 reflections	(Δ/σ)_max_ = 0.001
110 parameters	Δρ_max_ = 0.27 e Å^−^^3^
0 restraints	Δρ_min_ = −0.17 e Å^−^^3^

### Special details

**Table:**

Geometry. All s.u.'s (except the s.u. in the dihedral angle between two l.s. planes) are estimated using the full covariance matrix. The cell s.u.'s are taken into account individually in the estimation of s.u.'s in distances, angles and torsion angles; correlations between s.u.'s in cell parameters are only used when they are defined by crystal symmetry. An approximate (isotropic) treatment of cell s.u.'s is used for estimating s.u.'s involving l.s. planes.
Refinement. Refinement of F^2^ against ALL reflections. The weighted R-factor wR and goodness of fit S are based on F^2^, conventional R-factors R are based on F, with F set to zero for negative F^2^. The threshold expression of F^2^ > 2σ(F^2^) is used only for calculating R-factors(gt) etc. and is not relevant to the choice of reflections for refinement. R-factors based on F^2^ are statistically about twice as large as those based on F, and R- factors based on ALL data will be even larger.

### Fractional atomic coordinates and isotropic or equivalent isotropic displacement parameters (Å^2^)

**Table:**

	*x*	*y*	*z*	*U*_iso_*/*U*_eq_	
N1	0.09331 (7)	0.2065 (2)	0.61299 (9)	0.0337 (3)	
H1A	0.0694	0.3349	0.6498	0.051*	
H1B	0.0653	0.0629	0.6318	0.051*	
H1C	0.0824	0.2324	0.5410	0.051*	
C1	0.24993 (9)	0.0070 (3)	0.58382 (10)	0.0327 (3)	
C2	0.20875 (10)	−0.1621 (3)	0.50320 (11)	0.0386 (3)	
H2A	0.1430	−0.1576	0.4850	0.046*	
C3	0.26516 (12)	−0.3317 (3)	0.45199 (13)	0.0481 (4)	
H3A	0.2376	−0.4417	0.3993	0.058*	
C4	0.36465 (12)	−0.3402 (4)	0.47879 (14)	0.0540 (4)	
H4A	0.4024	−0.4558	0.4434	0.065*	
C5	0.40605 (11)	−0.1815 (4)	0.55573 (14)	0.0500 (4)	
H5A	0.4719	−0.1898	0.5723	0.060*	
C6	0.35070 (9)	−0.0034 (3)	0.61123 (11)	0.0385 (3)	
C7	0.39198 (10)	0.1629 (4)	0.69232 (13)	0.0489 (4)	
H7A	0.4576	0.1557	0.7106	0.059*	
C8	0.33727 (11)	0.3331 (4)	0.74393 (13)	0.0493 (4)	
H8A	0.3658	0.4407	0.7971	0.059*	
C9	0.23783 (10)	0.3478 (3)	0.71757 (11)	0.0390 (3)	
H9A	0.2006	0.4647	0.7528	0.047*	
C10	0.19664 (9)	0.1892 (3)	0.63994 (10)	0.0310 (3)	
Cl1	0.02807 (2)	0.28819 (7)	0.36504 (2)	0.03865 (12)	

### Atomic displacement parameters (Å^2^)

**Table:**

	*U*^11^	*U*^22^	*U*^33^	*U*^12^	*U*^13^	*U*^23^
N1	0.0316 (5)	0.0390 (7)	0.0306 (5)	0.0057 (5)	0.0026 (4)	−0.0010 (5)
C1	0.0331 (6)	0.0343 (8)	0.0309 (5)	0.0033 (6)	0.0033 (4)	0.0064 (6)
C2	0.0390 (6)	0.0398 (9)	0.0370 (6)	0.0039 (6)	0.0040 (5)	−0.0009 (6)
C3	0.0569 (9)	0.0442 (10)	0.0438 (7)	0.0070 (8)	0.0080 (6)	−0.0054 (7)
C4	0.0563 (9)	0.0516 (11)	0.0557 (9)	0.0219 (9)	0.0159 (7)	0.0019 (8)
C5	0.0375 (7)	0.0562 (11)	0.0571 (9)	0.0142 (8)	0.0082 (6)	0.0076 (8)
C6	0.0329 (6)	0.0408 (9)	0.0419 (7)	0.0037 (6)	0.0032 (5)	0.0084 (6)
C7	0.0338 (6)	0.0589 (11)	0.0530 (8)	−0.0028 (7)	−0.0045 (6)	0.0052 (8)
C8	0.0438 (8)	0.0560 (11)	0.0471 (8)	−0.0101 (8)	−0.0048 (6)	−0.0070 (8)
C9	0.0411 (7)	0.0398 (8)	0.0362 (6)	0.0003 (7)	0.0034 (5)	−0.0025 (6)
C10	0.0311 (5)	0.0334 (7)	0.0286 (5)	0.0024 (6)	0.0026 (4)	0.0042 (5)
Cl1	0.04571 (19)	0.0405 (2)	0.03008 (16)	0.00534 (16)	0.00533 (12)	0.00064 (14)

### Geometric parameters (Å, °)

**Table:**

N1—C10	1.4617 (16)	C4—C5	1.357 (3)
N1—H1A	0.8900	C4—H4A	0.9300
N1—H1B	0.8900	C5—C6	1.417 (2)
N1—H1C	0.8900	C5—H5A	0.9300
C1—C10	1.4189 (19)	C6—C7	1.415 (2)
C1—C2	1.420 (2)	C7—C8	1.360 (2)
C1—C6	1.4260 (17)	C7—H7A	0.9300
C2—C3	1.370 (2)	C8—C9	1.407 (2)
C2—H2A	0.9300	C8—H8A	0.9300
C3—C4	1.407 (2)	C9—C10	1.361 (2)
C3—H3A	0.9300	C9—H9A	0.9300
			
C10—N1—H1A	109.5	C4—C5—C6	121.17 (14)
C10—N1—H1B	109.5	C4—C5—H5A	119.4
H1A—N1—H1B	109.5	C6—C5—H5A	119.4
C10—N1—H1C	109.5	C7—C6—C5	122.29 (14)
H1A—N1—H1C	109.5	C7—C6—C1	119.33 (14)
H1B—N1—H1C	109.5	C5—C6—C1	118.38 (14)
C10—C1—C2	123.87 (12)	C8—C7—C6	121.10 (14)
C10—C1—C6	117.05 (13)	C8—C7—H7A	119.5
C2—C1—C6	119.08 (13)	C6—C7—H7A	119.5
C3—C2—C1	120.43 (13)	C7—C8—C9	120.51 (15)
C3—C2—H2A	119.8	C7—C8—H8A	119.7
C1—C2—H2A	119.8	C9—C8—H8A	119.7
C2—C3—C4	120.31 (16)	C10—C9—C8	119.33 (14)
C2—C3—H3A	119.8	C10—C9—H9A	120.3
C4—C3—H3A	119.8	C8—C9—H9A	120.3
C5—C4—C3	120.62 (15)	C9—C10—C1	122.68 (12)
C5—C4—H4A	119.7	C9—C10—N1	118.88 (12)
C3—C4—H4A	119.7	C1—C10—N1	118.44 (12)
			
C10—C1—C2—C3	179.52 (14)	C5—C6—C7—C8	179.64 (16)
C6—C1—C2—C3	−0.4 (2)	C1—C6—C7—C8	−0.4 (2)
C1—C2—C3—C4	0.0 (2)	C6—C7—C8—C9	−0.1 (3)
C2—C3—C4—C5	0.2 (3)	C7—C8—C9—C10	0.2 (2)
C3—C4—C5—C6	0.1 (3)	C8—C9—C10—C1	0.2 (2)
C4—C5—C6—C7	179.52 (17)	C8—C9—C10—N1	−179.85 (13)
C4—C5—C6—C1	−0.4 (2)	C2—C1—C10—C9	179.46 (14)
C10—C1—C6—C7	0.7 (2)	C6—C1—C10—C9	−0.6 (2)
C2—C1—C6—C7	−179.35 (14)	C2—C1—C10—N1	−0.53 (19)
C10—C1—C6—C5	−179.33 (13)	C6—C1—C10—N1	179.41 (12)
C2—C1—C6—C5	0.6 (2)		

### Hydrogen-bond geometry (Å, °)

**Table:**

*D*—H···*A*	*D*—H	H···*A*	*D*···*A*	*D*—H···*A*
N1—H1A···Cl1^i^	0.89	2.41	3.1824 (11)	145.
N1—H1B···Cl1^ii^	0.89	2.27	3.1355 (11)	164.
N1—H1C···Cl1	0.89	2.24	3.1225 (11)	170.

Symmetry codes: (i) −*x*, −*y*+1, −*z*+1; (ii) −*x*, −*y*, −*z*+1.
